# Preservation of Microalgae, Lignocellulosic Biomass Blends by Ensiling to Enable Consistent Year-Round Feedstock Supply for Thermochemical Conversion to Biofuels

**DOI:** 10.3389/fbioe.2020.00316

**Published:** 2020-04-15

**Authors:** Bradley D. Wahlen, Lynn M. Wendt, Austin Murphy, Vicki S. Thompson, Damon S. Hartley, Thomas Dempster, Henri Gerken

**Affiliations:** ^1^Biological Processing, Idaho National Laboratory, Idaho Falls, ID, United States; ^2^Biofuels Center of Excellence, Santa Fe Community College, Santa Fe, NM, United States; ^3^Arizona Center for Algae Technology and Innovation, Arizona State University, Tempe, AZ, United States

**Keywords:** microalgae, biofuels, ensiling, hydrothermal liquefaction, resource assessment, preservation

## Abstract

Seasonal variation in microalgae productivity is a significant barrier to economical production of algae biofuels and chemicals. Summer production can be 3–5 times higher than in the winter resulting in uneven feedstock supplies at algae biorefineries. A portion of the summer production must be preserved for conversion in the winter in order to maintain a biorefinery running at capacity. Ensiling, a preservation process that utilizes lactic acid fermentation to limit microbial degradation, has been demonstrated to successfully stabilize algae biomass (20% solids) and algae-lignocellulosic blends (40% algae-60% lignocellulosic biomass, dry basis) for over 6 months, resulting in fuel production cost savings with fewer emissions. Preservation of algae as blends could be beneficial to biorefineries that utilize thermochemical approaches to fuel production as co-processing of algae and lignocellulosic biomass has been observed to enhance biocrude yield and improve oil quality. This study conducts a resource assessment of biomass residues in the southern United States to identify materials available during peak algae productivity and in sufficient quantity to meet the algae storage needs of an algae biofuel industry. Eight feedstocks met the quantity threshold but only three, distillers grains, haylage, and yard waste, were also available in season. Storage experiments utilizing both freshwater and marine strains of microalgae – *Scenedesmus acutus*, *Chlorella vulgaris*, *Chlorella zofingiensis*, *Nannochloropsis gaditana*, and *Porphyridium purpureum* – and yard waste were conducted for 30 days. Storage losses were less than 10% in all but one case, and the pH of all but one blend was reduced to less than 4.7, indicating that yard waste is a suitable feedstock for blending with algae prior to storage. To better understand whether the benefits to conversion realized by processing blends might be affected by storage, elemental analysis and bomb calorimetry of pre- and post-storage algae-yard waste blends were conducted to characterize changes occurring during storage. Storing algae biomass as blends with lignocellulosic biomass could be an effective method of mitigating seasonal variability in algae biomass production while retaining the synergistic effect of co-processing algae blends in thermochemical conversion.

## Introduction

Microalgae are a promising feedstock for biofuel production due to their high energy content relative to other feedstocks, their rapid growth rate, and ability to be cultivated on marginal lands using non-potable water (e.g., brackish water and seawater) (Wijffels and Barbosa, 2010; Wijffels et al., 2010; Williams and Laurens, 2010). Yet, despite these benefits, challenges to commercialization of algae for fuel production remain (Greenwell et al., 2010; Bull and Collins, 2012; Day et al., 2012). Providing a consistent year-round supply to an algae biorefinery is a recognized barrier to economically produced algae biofuels (Davis R. et al., 2014). Like most crops, algae biomass production varies seasonally with maximum yields occurring during the summer months (June–August), where production can be 3–5 times greater than that achieved in the winter (Davis R. E. et al., 2014).

Variability in algae productivity complicates the sizing of downstream conversion facilities (Davis R. et al., 2014) since biorefineries sized to accommodate summer productivity would be underutilized in the winter. Design cases for the production of biofuels from algae biomass sponsored by the United States Department of Energy Bioenergy Technologies Office (DOE-BETO) mitigate for seasonal variability in algae production by designing conversion facilities to accommodate spring biomass production rates, requiring the preservation of excess algal biomass produced in the summer for conversion in the winter (Davis R. et al., 2014; Jones et al., 2014). In this manner conversion facilities can operate at capacity year-round. Long-term preservation of algae biomass, however, is challenging due to the high moisture content (80%, wet basis) of harvested algae biomass. Drying is a common approach to preserving high moisture plant material but the algae biomass (20% solids) rheology and high moisture content (80%, wet basis) make this both technically challenging and costly (Wahlen et al., 2017). Harvested microalgae biomass is also susceptible to microbial degradation and requires active storage solutions to limit biomass loss (Wendt et al., 2017a).

Ensiling is an alternative preservation strategy that does not require drying. Herbaceous biomass is regularly preserved through ensiling for the forage industry and can be used to stabilize high-moisture feedstock destined for bioenergy production (Wilkinson et al., 2003; Wendt et al., 2018). Oxygen-limited conditions in ensiling enable the fermentation of soluble sugars to organic acids, resulting in a lower pH that inhibits microbial activity (Rooke and Hatfield, 2003). Utilizing ensiling instead of drying as a preservation strategy for microalgal biomass can reduce the cost of fuel production by $0.32 per gallon of gasoline equivalent (GGE) (Wendt et al., 2019).

Thermochemical conversion of algae blended with lignocellulosic biomass to fuels by hydrothermal liquefaction (HTL) has many benefits that could serve to reduce the cost of producing fuel from microalgae biomass (Jarvis et al., 2018). Jarvis et al. (2018) noted that bio-oil resulting from HTL processing of algae-herbaceous blends had novel compounds that were not present in either feedstock processed alone. Blend bio-oil also contained less N and less O than the biocrudes derived from algae and lignocellulosic biomass, respectively. Co-processing algae and lignocellulosic biomass also had operational benefits. When processing lignocellulosic material, a buffer such as Na_2_CO_3_ is typically co-fed to neutralize acidic products and obtain a neutral bio-oil. When lignocellulosic biomass was processed along with algae, a neutral bio-oil was produced that did not require addition of the buffer. Surprisingly, the authors of this study also noted that processing algae-lignocellulosic blends had a synergistic effect on biocrude yield; more biocrude was produced from blends than from either feedstock by itself (Jarvis et al., 2018). Preservation of algae blended with lignocellulosic biomass will be essential to ensuring that the benefits of algae blends to HTL conversion are realized year-round.

Ensiling has been shown to be an effective approach to stabilizing microalgae blended with corn stover (Wendt et al., 2017a). When blends containing 40% microalgae biomass and 60% corn stover (dry basis) were inoculated with *Lactobacillus acidophilus* storage losses were limited to <8% dry matter after 35 days in storage (Wendt et al., 2017a). Furthermore, ensiling was estimated to be only 65% of the cost of drying while requiring 10% as much energy and reducing greenhouse gas emissions by as much as 75% (Wendt et al., 2017b). Ensiling microalgae-lignocellulosic biomass blends could be an effective approach to preserving material for the year-round operation of an HTL facility.

Although algae blended with corn stover has been shown to be stably preserved through ensiling for extended periods of time, corn stover is not an ideal herbaceous blendstock because the two crops do not overlap in their season of production in the majority of the United States. Corn stover is available in the fall, when algae biomass is expected to be utilized in conversion processes as it is produced. Therefore, an alternative crop residue, available during the summer months, is needed to enable storage of excess algae as blends with herbaceous biomass. In this study, a resource assessment of biomass residues available in the southern United States was conducted to identify underutilized biomass that is widely available during the precise time when it is needed and yard waste was identified as a likely candidate. Storage studies were then conducted with multiple strains of algae blended with yard waste to determine the suitability of this approach. Stored algae/yard waste blends were then further characterized to determine how compositional changes occurring in storage might affect HTL conversion of algae-yard waste blends.

## Materials and Methods

### Materials

Algae cultivation of *Scenedesmus acutus*, *Nannochloropsis gaditana*, *Chlorella zofingiensis*, *Chlorella vulgaris*, and *Porphyridium purpureum* was performed at the Arizona Center for Algae Technology and Innovation in Mesa, AZ, in a containment greenhouse. *S. acutus* LRB0401 was inoculated at 0.05 g/L and grown in BG-11 medium. Algae was cultured in 110 L vertical flat panel photobioreactors with a 2-in. light path using natural lighting (natural diurnal light and dark periods). High temperatures averaged 20°C and low temperatures averaged 7°C during both batch runs. Each batch culture was grown over a 3-week period and harvested when culture density reached 3 g/L. The algae biomass was dewatered at 1800 × g through Lavin 20–1160 V Centrifuges (AML Industries, Inc., Warren, OH, United States) with a flow rate of approximately 2 L/min. Dewatered algae were placed into Ziploc^®^ bags, stored in a cooler on ice, and shipped overnight to Idaho National Laboratory. The other strains were grown in a similar manner. Media for *N. gaditana* and *P. purpureum* was adjusted to 35 g/L salt using Oceanic Sea Salt. Yard waste (grass clippings and leaves) was collected fresh and frozen prior to size reduction with a Wiley mill (model 4, Thomas, Swedesboro, NJ, United States) to pass through 6 mm screen. Yard waste remained frozen during size reduction.

### Resource Assessment

This resource assessment provides an estimate of the feedstock inventories for the southeastern and southwestern regions of the United States. The purpose of the assessment is to provide insight into the types of feedstocks that may be available in each region but does not make assertions about availability or prices needed to divert the feedstocks from current uses. The data for the crops and crop residues came from the 2012 Census of Agriculture (Vilsack, 2014). When feedstock information was not directly available from the source, a residue-to-product ratio was used to estimate the quantity of residues available based on the primary product yield (Koopmans and Koppejan, 1997). Distiller’s grains inventories were estimated based on ethanol plant location and production; the locations and production of currently operation ethanol plants were taken from Ethanol Producer Magazine (Ethanol Producer Magazine, 2016) with a factor of 17 dry tons of distillers grain per gallon of production. The production of MSW yard waste is based on population. The average value of yard waste produced per person per year was defined from a sample of published location specific waste generation reports (cited in Supplementary Material). The average value was then multiplied by the county population to estimate the inventory of yard waste. The quantity of each feedstock was then georeferenced to a county in ArcGis 10.2.3 to produce spatial coverages for the feedstocks (Supplementary Figures 1–18).

### Storage Experiments

Storage studies were conducted in 4 oz (118 mL) or 16 oz (473 mL) air-tight mason jars (Ball Mason Jars, Newell Brands, Atlanta Georgia). Gas collection was accommodated by fitting standard canning lids (Ball Mason Jars, Newell Brands, Atlanta Georgia) with bulkhead fittings (P/N SS-400-61, Swagelok, Solon, OH, United States). Rubber gasket material and stainless-steel washers were used to seal the bulkhead fitting to the lid. A quarter-turn plug valve (P/N B-4P4T, Swagelok, Solon, OH, United States) was connected to the bulkhead fitting with 1/4′′ OD stainless steel tubing to facilitate reactor headspace gas exchange with nitrogen at the beginning of storage studies. Fermentation gas was collected in foil gas collection bags (FlexFoil, P/N 262-01, SKC, Inc., Eighty Four, PA, United States) connected to the plug valve with either silicon tubing (P/N EW-96410-16, Cole-Parmer, Vernon Hills, IL, United States) or C-flex ULTRA tubing (P/N EW-06434-16, Cole-Parmer, Vernon Hills, IL, United States). Microalgae biomass (20% solids) and yard waste was mixed together using a handheld kitchen blender for approximately 5 min. Algae-yard waste blended material was then packed into pre-weighed jars, pre-weighed lids were tightened and the assembled jar containing biomass was weighed again. The biomass loading varied with experiment depending on the amount of algae available from 16 to 36 g (dry basis) in 118 mL jars and 106 g (dry basis) in 473 mL jars. Jars were then made anaerobic by subjecting the headspace to vacuum and then nitrogen gas, repeating the process three times. Once anaerobic, jars were fitted with a gas collection bag and placed in the dark at room temperature for 30 days.

At the conclusion of the storage period jars with and without lids were weighed again. Moisture content of initial and stored material was determined gravimetrically after drying at 105°C until reaching a constant weight. Dry matter loss for each storage replicate is reported as a percentage of the initial material according to Eq. (1):

%⁢d⁢r⁢y⁢m⁢a⁢t⁢t⁢e⁢r⁢l⁢o⁢s⁢s

$$\;=\left[\frac{\left(I\,n\,i\,t\,i\,a\,l\,d\,r\,y\,m\,a\,t\,e\,r\,i\,a\,l\,\left(g\right)-F\,i\,n\,a\,l\,d\,r\,y\,m\,a\,t\,e\,r\,i\,a\,l\,\left(g\right)\right)}{I\,n\,i\,t\,i\,a\,l\,d\,r\,y\,m\,a\,t\,e\,r\,i\,a\,l\,\left(g\right)}\right]$$

(1)   *100

Stored biomass was removed from each jar, sampled for moisture and organic acid content and frozen at −20°C until used for further analysis.

### Analysis of Fermentation Products

Gases and organic acids produced during the ensiling process were collected and analyzed as previously described (Wendt et al., 2017a). Briefly, the total volume of gases collected in gas sampling bags over the course of the storage period was measured and the composition of the gas (CH_4_, CO, H_2_, N_2_, O_2_, and CO_2_) was determined by gas chromatography as previously described (Wendt et al., 2017a). The quantity of nine organic acids (succinic acid, lactic acid, formic acid, acetic acid, propionic acid, isobutyric acid, butyric acid, isovaleric acid, and valeric acid) from each storage replicate were measured by HPLC as previously described (Wendt et al., 2017a). The HPLC detector was calibrated with standards at five concentration levels (P/N 95917, Absolute Standards, Inc., Hamden, CT, United States). Duplicate samples from each storage replicate were measured in duplicate by HPLC.

### Elemental Analysis

Biomass from the larger-scale (∼500 mL) storage study of *S. acutus* microalgae biomass blended with yard waste was analyzed for C, H, N, O, and S content and for energy density (i.e., calorimetry). This was done for both initial materials (yard waste, *S. acutus* biomass, the unstored blend) and each 30-day storage replicate. The yard waste, initial blend and stored blends were prepared for analysis by first drying at 105°C followed by size reduction to a top size of 0.2 mm in a Retsch ultra centrifugal mill (Retsch, Haan, Germany). The *S. acutus* initial staring material was first freeze-dried and then ground to a fine powder by mortar and pestle. Elemental analysis (C, H, N, and S) was accomplished using a LECO TruSpec CHN with S add-on module (LECO, St. Joseph, MI, United States) following ASTM D5373-10 (CHN) and ASTM D 4239-10 (S) (ASTM, 2010a, b). Oxygen was determined by difference. Samples were analyzed in triplicate.

## Results and Discussion

### Resource Assessment

The United States DOE-BETO has established a milestone within their Multi-Year Program Plan (MYPP) to model the sustainable supply of 20,000,000 tonnes (22,046,000 United States ton) of algal biomass annually by 2022 (DOE-BETO, 2016). Based on current design cases, a portion of algal biomass, which amounts to ∼ 6.5% of the total annual algal biomass production, will be produced in excess of conversion capacity during productive summer months and will need to be preserved for use later in the year (Davis R. et al., 2014). This equates to 1,300,000 tonnes (1,143,000 United States ton) of algal biomass. To achieve a blending ratio of 40% algae and 60% lignocellulosic biomass for preservation through ensiling, 1,950,000 tonnes (2,149,507 United States ton) of wet herbaceous biomass needs to be identified to preserve excess biomass from 20,000,000 tonnes algal biomass produced annually.

**FIGURE 1:**
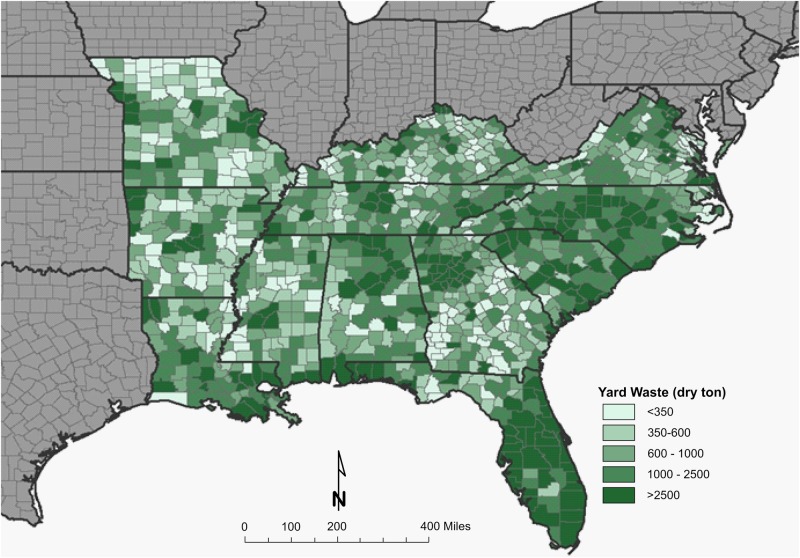
County-level resolution of annual inventory of yard waste in the Southeastern United States.

**FIGURE 2:**
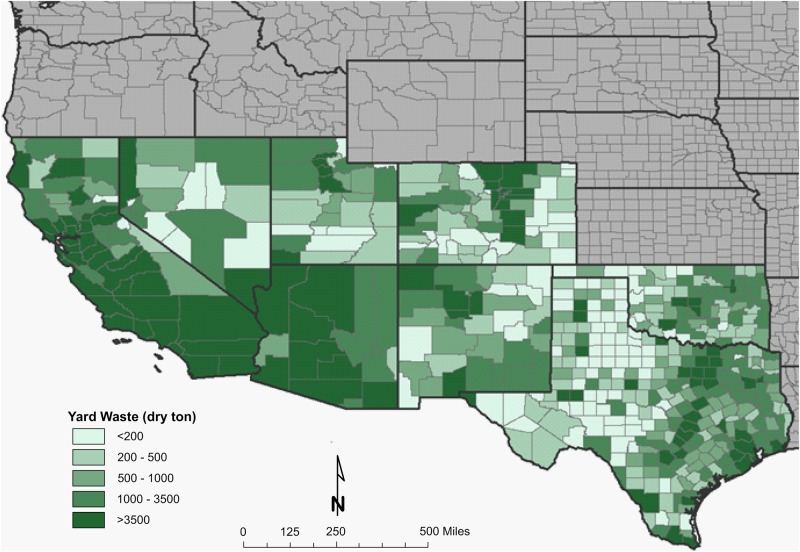
County-level resolution of annual inventory of yard waste in the Southwestern United States.

A resource assessment of crops and crop residues suitable for blending with algae and ensiling was conducted in the southern United States, an area expected to be productive for microalgae cultivation (Wigmosta et al., 2011). A total of eight feedstocks were identified that are currently being produced across the southern United States in sufficient quantity to be blended with 1,300,000 tonnes of algal biomass and preserved through ensiling (Table 1). Corn stover, energy/sugar cane and rice straw are the most abundant crop residues identified in this assessment, however, their availability does not overlap with the most productive months for algae (June–August). Yard waste, haylage and distillers grains are each available in sufficient quantity for blending and storing in the season required. Both haylage [$110–$220 ton^–1^ (USDA, 2018)] and distiller’s grains [$130-$175 ton^–1^ (USDA, 2020)] have high feedstock costs because of their value as livestock feed. Yard waste, however, has limited utility and its disposal is often accompanied by a tipping fee, leading to low feedstock cost [$64 ton^–1^ (Roni et al., 2019)]. Therefore, yard waste was selected as a representative feedstock for storage studies to understand how storage might impact the thermochemical conversion of the blend. Figures 1, 2 display county level annual availability of yard waste. Maps (Supplementary Figures 1–17) and feedstock-specific information are available in the Supplementary Material for each feedstock included in the resource assessment.

**TABLE 1:** Resource assessment of biomass residues available annually in the southern United States.

	**Southwestern United States**		**Southeastern United States**	
		
**Resource**	**Period of availability**	**Estimated annual inventory (ton)**	**Period of availability**	**Estimated annual inventory (ton)**
Corn Stover	August–November	11,187,082	July–October	27,958,773
Cotton stalks	October–December	4,064,226	September–November	4,174,541
Peanut hay	October–November	462,923	September–October	4,383,210
Rice straw	August–September	2,756,610	August–October	7,157,144
Sorghum	August–October	1,256,652	August–October	776,201
Haylage	April–September	2,866,954	April–September	934,803
Distillers grains	Continuous	2,496,000	Continuous	2,064,000
Sugar cane/energy cane*	November–March	1,401,926	October–March	27,380,199
Yard waste	April–September	2,588,903	April–September	2,439,955

*Annual inventory considers the bagasse of sugar cane/energy cane.

The quality of the feedstocks included in the resource assessment and their suitability for HTL conversion varied from one feedstock to another. Elemental analysis of feedstocks measuring the carbon, hydrogen, nitrogen, sulfur, and oxygen content can be a good indicator of the quality of a material for thermochemical conversion. Feedstocks with higher amounts of carbon and hydrogen will have greater energy content, while the presence of nitrogen and oxygen decrease the energy content. The elemental analysis of feedstocks included in the resource assessment are listed in Table 2, with the exception of haylage and peanut hay. Cotton stalks had the highest carbon content of any of the herbaceous feedstocks followed by distiller’s grains (49.3% and 48.8%, respectively). The energy content of distiller’s grains, however, was higher (21.2 MJ/kg vs. 18.4 MJ/kg) due to its lower oxygen content compared to cottons stalks (34% vs. 43%) and was the highest of any of the feedstocks.

**TABLE 2:** Elemental composition of herbaceous feedstocks included in geographical resource assessment.

**Material**	**Ash (%, db)**	**C (%, db)**	**H (%, db)**	**N (%, db)**	**O (%, db)**	**S (%, db)**	**HHV (MJ/kg)**	**References**
Corn stover	4.7	47.9	5.9	1.7	38.6	0.18	19.8*	Wendt et al., 2017a
Cotton stalks	2.7	49.3	6.3	0.8	43.5	ND	18.4	Fu et al., 2012
Peanut Hay	–	–	–	–	–	–	–	Data not available
Rice straw	13.9	44.2	6.2	0.8	48.8	ND	17.4*	Worasuwannarak et al., 2007
Sorghum	4.6	41.3	5.4	1.3	52.0	ND	16.3	Yue et al., 2018
Haylage	–	–	–	–	–	–	–	Data not available
Distillers grains	ND	48.8	6.6	5.4	34.1	ND	21.2*	Wang and Brown, 2014
Sugar cane bagasse	1.6	45.5	5.6	0.8	48.1	ND	17.5*	Waheed and Williams, 2013
Yard waste	9.7	45.2	5.9	3.5	32.7	0.23	19.1	This study

db, dry basis. *Higher heating value (HHV) was calculated by the method of Channiwala and Parikh (2002). ND, not determined.

Yard waste, another feedstock whose season of availability coincided with peak algae production, had an energy content (19.1 MJ/kg) higher than many of the other feedstocks in the resource assessment. In addition, the low cost of yard waste makes it an attractive feedstock to blend with algae prior to storage. The disposal of yard waste often carries a tipping fee to the landfill or disposal site, causing the cost of obtaining this biomass to be very low (Roni et al., 2019). However, one drawback of yard waste as a feedstock is the ash content, which represents a fraction of the biomass that cannot contribute to biofuel production and can affect the operation of a biorefinery (Lacey et al., 2018). Blending has been previously shown to be an effective approach to reducing the ash content of a feedstock, such as yard waste (Thompson et al., 2019). The anticipated amount of algae produced in excess of conversion capacity during the summer amounts to only 6.5% of the total annual algae production. Therefore, when stored algae-lignocellulosic blends are needed to fill gaps in algae production, they will be blended with freshly harvested algae and other seasonally available biomass residues, effectively diluting the amount of ash contributed by yard waste. To take advantage of its low cost, in-season availability, and higher energy content, yard waste was selected as the blending agent for storage experiments.

### Storage Performance

Storage studies of algae-yard waste blends were conducted with biomass from multiple strains of algae for a period of 30 days in anaerobic conditions. Both freshwater (*S. acutus*, *C. zofingiensis*, and *C. vulgaris*) and saltwater (*N. gaditana* and *P. purpureum*) strains were mixed with yard waste and evaluated for stability in storage. Dry matter loss, a measurement of how much material is consumed in storage by biological processes, ranged from a low of 4.0% (dry basis, db) in the case of *S. acutus* at the 500 mL scale to a high of 12.8% (db) occurring in stored *N. gaditana*-yard waste blends (Table 3). All but *N. gaditana* resulted in losses lower than 10% (db). The final pH of stored biomass ranged from 3.90 to 7.05. Generally, pH below 4.5 was indicative of low dry matter loss, the exception being *C. vulgaris*-yard waste blend, which achieved the lowest pH (3.90) but had the second highest dry matter loss (9.7). One explanation could be the large total organic acid production occurring in *C. vulgaris*-yard waste blends, which produced the most organic acids in storage (see Section “Organic Acid Production”). The formation of some organic acids is accompanied by CO_2_ production and therefore loss of biomass. Lactic acid fermentation, where there are two pathways for production, is a good example. Homolactic fermentation produces only lactic acid with no loss of carbon, whereas heterolactic fermentation produces acetic acid and CO_2_ in addition to lactic acid (McGechen, 1990).

**TABLE 3:** Storage performance of algae blended with yard waste (40% algae:60% yard waste).

**Organism**	**Scale (mL)**	**Dry matter loss (%, db)**	**pH**	**Lactic acid (%, db)**	**Organic acid (%, db)**	**CO_2_ (g/kg, db)**
*Scenedesmus acutus*	100	4.8 ± 0.8	3.98 ± 0.01	8.9 ± 0.3	16.3 ± 0.5	4.6 ± 0.4
*Scenedesmus acutus*	500	4.0 ± 0.3	3.98 ± 0.3	9.9 ± 0.1	20.8 ± 0.4	5.4 ± 0.4
*Nannochloropsis gaditana*	100	12.8 ± 1.2	7.05 ± 0.13	5.6 ± 1.1	15.4 ± 0.8	41.0 ± 2.5
*Chlorella zofingiensis*	100	5.6 ± 0.7	4.09 ± 0.04	10.8 ± 0.6	19.9 ± 1.1	0.9 ± 0.8
*Chlorella vulgaris*	100	9.7 ± 0.6	3.90 ± 0.01	11.6 ± 0.4	21.2 ± 0.0	0
*Porphyridium purpureum*	100	6.5 ± 2.6	4.74 ± 0.04	8.8 ± 0.3	19.8 ± 1.7	13.4 ± 4.1

*db, dry basis. values are the average of triplicate measurements and the variation is the standard deviation.*

CO_2_ measurement in algae-yard waste storage experiments has proved to be challenging. The volume of total gas evolution was measured for each storage replicate and carbon dioxide was quantified. The highest measured production of CO_2_ occurred in the *N. gaditana* blends (41 ± 2.5) where the greatest dry matter loss was also observed. Measured carbon dioxide evolution alone does not account for total dry matter loss. For *N. gaditana* the dry matter loss experienced was 12.8% of the initial dry matter, while CO_2_ evolution accounted for only 32% of that loss. The ratio of CO_2_ to total loss was highest in *N. gaditana*. For the *C. vulgaris*-yard waste blend no gas evolution was measured at all, despite having the second greatest dry matter loss. It has been noted in other silage studies that CO_2_ measurement is difficult in laboratory-scale silos and often results in large variation among replicates (El Hag et al., 1982; Weinberg et al., 1995). In the present study, we have observed algae-yard waste blends expand in storage due to gas production, causing some reactors to buckle and fail. The algae-yard waste blends tend to trap gases rather than allowing them to release. In some experiments a greater headspace was left to accommodate this expansion, and nitrogen gas-vacuum cycles were used to purge the jars of oxygen and establish an anaerobic environment. The addition of nitrogen gas to laboratory reactors further complicates the measurement of CO_2_.

### Organic Acid Production

**FIGURE 3:**
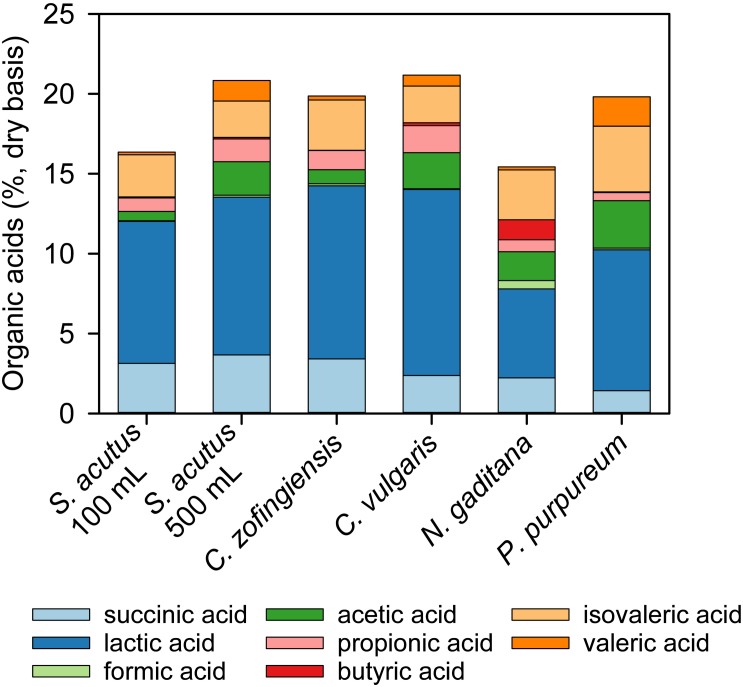
Composition of organic acids produced during wet anaerobic storage of algae-yard waste blends. Yard waste was blended with microalgae biomass from multiple species and stored anaerobically for 30 days in 100 mL volumes unless otherwise indicated. All blends contained 60% yard waste and 40% algae biomass on a dry material basis.

The content and composition of organic acids present in stored algae-yard waste blends varied among the different strains of microalgae evaluated (Table 3 and Figure 3). The *C. vulgaris*-yard waste blend generated the most organic acids in storage (21.2%, db) and *N. gaditana* the lowest (15.4%, db). Lactic acid was the primary component of total organic acids in each stored blend and was greater than 50% of total organic acids in all but three cases (*N. gaditana* 36%, *P. purpureum* 44%, and *S. acutus*, 500 mL, 49%). Butyric acid was present in each stored blend at less than 1% of total organic acids except for stored the *N. gaditana*-yard waste blend where it made up 8% of total organic acids. Succinic and acetic acids also comprised substantial proportions of total organic acids (7–19% and 3–15%, respectively).

The presence of organic acids in the post-storage biomass could have positive benefits in HTL conversion. Ross et al. (2010) explored the use of alkali and organic acids as catalysts in the HTL processing of microalgae. They found that biocrude yield was increased with organic acid catalysts relative to the alkali. Organic acid catalysts also affected the quality of the biocrude by increasing the size distribution of biocrude molecules with greater lower molecular weight compounds than observed with the alkali catalysts. This resulted in a biocrude with a lower boiling point and improved flow properties (Ross et al., 2010). Although Ross et al. (2010) performed their study with formic and acetic acids, the presence of longer chain organic acids (e.g., lactic acid, propionic acid) in the stored algae-yard waste blends could similarly benefit biocrude yield and quality.

### Elemental Composition

Often proximate and ultimate analysis is used to determine the suitability of materials for thermochemical conversion to oils in processes such as HTL. The blend of *S. acutus* and yard waste was selected as a representative algae-yard waste blend to determine whether these types of blends would be suitable for HTL conversion and what impacts anaerobic storage might have on the suitability of this material. To accommodate the analysis, this particular blend was prepared in sufficient quantity and stored at a greater scale compared to the other algae-yard waste blends evaluated in storage only. Table 4 lists the ash content, elemental composition and energy density for pre-storage yard waste, *S. acutus* biomass and the two blended together at a 60:40 ratio (yard waste: *S. acutus*) and for the post-storage blend.

**TABLE 4:** Elemental composition of yard waste, algae and algae-yard waste blends before anaerobic storage and algae-yard waste blends after 30 days anaerobic storage.

**Material**	**Time stored (days)**	**Ash (%, db)**	**C (%, db)**	**H (%, db)**	**N (%, db)**	**O (%, db)**	**S (%, db)**	**HHV (MJ/kg)**
Yard Waste	0	9.74 ± 0.03	45.25 ± 0.09	5.95 ± 0.10	3.52 ± 0.02	35.31 ± 0.21	0.23 ± 0.01	19.1 ± 0.1
*S. acutus*, 20% solids	0	4.46 ± 0.00	52.14 ± 0.02	7.27 ± 0.02	8.42 ± 0.05	27.43 ± 0.18	0.29 ± 0.04	23.9 ± 0.0
*S. acutus*-yard waste blend	0	6.96 ± 0.16	48.08 ± 0.23	6.55 ± 0.16	5.19 ± 0.08*	33.02 ± 0.45	0.19 ± 0.01	21.2 ± 0.1
*S. acutus*-yard waste blend	30	7.03 ± 0.01	48.14 ± 0.03	6.64 ± 0.12	5.30 ± 0.02*	32.70 ± 0.06	0.19 ± 0.02	21.2 ± 0.2

*db, dry basis. *S. acutus*-yard waste blends contained 40% algae-60% yard waste on a dry basis. Values are the average of triplicate measurements and the variation is the standard deviation. Storage experiments were conducted in triplicate. Post-storage material was combined to form a composite sample which was then measured in triplicate. Differences between the material properties of each material was determined by One Way ANOVA with pairwise comparison by the Holm–Sidak method (*p* < 0.05). Each property of the initial starting material (yard waste, *S. acutus* and *S. acutus*-yard waste blends) except sulfur content was different from one another. The lone difference between the *S. acutus* blend caused by storage is marked with an asterisk.*

Initial starting materials differed from one another in nearly every aspect. The ash content of the yard waste was 9.74% (db) compared to 4.46% (db) for algae biomass. Ash content of algae is likely to be highly variable between different species and different cultivation methods for a given species. Marine strains have higher ash content than freshwater strains due to the greater salt content of seawater. Strains cultivated in open raceways are likely to have higher ash content than strains cultivated in photobioreactors due to concentration of salts caused by evaporation and from soil particles entering from the external environment.

Algae typically contain higher proportions of protein and lipid than herbaceous biomass and less carbohydrates. This is reflected in the elemental composition of yard waste and *S. acutus* biomass. *S. acutus* biomass is more carbon (52%) and hydrogen (7%) rich compared with yard waste (45% and 6%, respectively) due to the lipid content of microalgae, and yard waste has a greater oxygen content due to its structural sugar content (cellulose and hemicellulose). Algae nitrogen content is substantially higher than that found in the yard waste (8.4% vs. 3.5%), likely due to differing concentrations of protein in the two materials. The blend of yard waste and algae (60:40) resulted in a material with properties consistent with the composition of the initial materials and their proportion in the final blend.

The energy content of the two initial unblended materials is consistent with their respective elemental compositions. The relationship between elemental composition and energy content is described by several equations (Channiwala and Parikh, 2002). Generally, carbon and hydrogen content correlate positively with energy content, while oxygen correlates negatively. The energy content reported in Table 4 is measured as described in the materials and methods and not calculated. Yard waste has a lower energy content than algae biomass (19.1 MJ/kg vs. 23.9 MJ/kg). Blending of the two materials results in a blend with energy content that is intermediate to the two initial feedstocks.

Post-storage algae-yard waste blends did not differ in elemental composition. A one-way ANOVA analysis did not find any differences in ash, C, H, O, or S content (*p* < 0.05). There was a small statistically significant difference in nitrogen content with the stored blend having a slightly higher nitrogen content. Though significant, this difference is likely inconsequential. As one would expect based on the correlation between elemental composition and energy content, calorimetry did not find any significant difference in the energy content of stored and unstored algae-yard waste blends.

## Conclusion

This study has identified eight crops or crop residues in the southern United States that could support the preservation needs of 20,000,000 metric tonnes of algal biomass annually. Although only distiller’s grains, haylage and yard waste were available when algae biomass production is maximal. Storage studies conducted with yard waste and several freshwater and marine strains of algae were successfully preserved over 30 days with all but one experiencing less than 10% dry matter loss and a final pH of less than 4.7. Elemental analysis of stored *S. acutus* blends demonstrated that the elemental composition and the higher heating values do not change significantly due to storage. This raises the possibility that fuel yield in HTL may also be unaffected by storage. The production of organic acids in storage significantly increases their presence in the biomass and their ultimate effect on HTL processing, biocrude yield and processing, is uncertain. Direct HTL processing of stored algae-lignocellulosic blends is needed to accurately assess the impact of storage on the yield of biocrude and the quality of final fuel.

## Data Availability Statement

The resource assessment for each feedstock and its source information is included in the article/Supplementary Material.

## Author Contributions

BW and LW designed the study. BW, LW, and AM conducted the storage experiments. VT and DH conducted the resource assessment. TD and HG provided algae biomass. BW drafted the manuscript with contributions from each co-author. All authors read and approved the final version of the manuscript.

## Conflict of Interest

The authors declare that the research was conducted in the absence of any commercial or financial relationships that could be construed as a potential conflict of interest.
