# Supplementary material for: Preservation of Microalgae, Lignocellulosic Biomass Blends by Ensiling to Enable Consistent Year-Round Feedstock Supply for Thermochemical Conversion to Biofuels
Source: Front Bioeng Biotechnol. 2020 Apr 15;8:316. doi: 10.3389/fbioe.2020.00316 (PMC7174550; doi:10.3389/fbioe.2020.00316)
Supplement: Supplementary file 1 [file Table_1.DOCX]

***Methods for Resource Assessment***

This resource assessment provides an estimate of the feedstock inventories for the southeastern and southwestern regions of the United States. The purpose of the assessment is to provide insight into the types of feedstocks that may be available in each region, but does not make assertions about availability or prices needed to divert the feedstocks from current uses. The data for the crops and crop residues came from the 2012 Census of Agriculture (Vilsack 2014). When feedstock information was not directly available from the source, a residue-to-product ratio was used to estimate the quantity of residues available based on the primary product yield (Koopmans and Koppejan 1997). For the non-agronomic crops, distillers grains and yard waste, the inventories were estimated base on ethanol plant location and production in the case of distillers grains and population for yard waste. The locations and production of currently operating ethanol plants were taken from Ethanol Producer Magazine (Ethanol Producer Magazine 2016), with a factor of 17 dry tons of distillers grain per gallon of production. The production of yard waste is based on population. The average value of yard waste produced per person per year was defined from a sample of published waste generation reports (R.W. Beck 200, DSM Environmental Services 2002, Cascadia Consulting Group, R.W. Beck et al. 2003, Cascadia Consulting Group, Sky Valley Associates et al. 2003, Franklin Associates 2003, Engineering Solutions and Design 2004, R.W. Beck 2005, RIDOP 2005, TCEQ 2006, Cascadia Consulting Group, DSM Environmental Services et al. 2007, Mid Atlantic Solid Waste Consultants 2007, Cascadia Consulting Group 2008, Midwest Assistance Program 2008, CDM 2009, Engineering Solutions and Design 2009, Cascadia Consulting Group 2010, Covanta Energy SEMASS and Mid Atlantic Solid Waste Consultants 2010, Covanta Energy Springfield and Mid Atlantic Solid Waste Consultants 2010, Florida Department of Environmental Protection 2010, MSWConsultants and Cascadia Consulting Group 2010, NYSDEC 2010, ODEQ 2010, SCS Engineers 2010, SDM Envrionmental Services, Cascadia Consulting Group et al. 2010, 2011, Mid Atlantic Solid Waste Consultants 2011, Abramowitz and Sun 2012, Criner 2012). The average value was then multiplied by the county population to estimate the inventory of yard waste. The quantity of each feedstock was then georeferenced to a county in ArcGis 10.2.3 to produce spatial coverages for the feedstocks.

***Corn Stover***

Corn stover, a by-product of corn grain production, is the highest volume of residue of all the major U.S. agricultural crops and has been increasing as the corn grain yield has increased (Jeschke and Heggenstaller 2012). Corn stover is harvested in two ways either as a dry material with a moisture content less than 20% or as a silage material with a moisture content of >40%. The harvest and collection of corn stover as a dry material is generally associated with the production of corn grain and occurs after the grain harvest. The harvest of the corn as silage generally occurs earlier in the year, approximately one month earlier than the grain harvest in the same area, before full development of the corn grain and while the moisture in the plant is relatively high.

*Southeastern U.S.:* In the southeastern US the production of corn is primarily located in the lower Ohio, Missouri and Mississippi River valleys. Additionally a secondary area of production is located along the coastal plains of Georgia, South Carolina, North Carolina and Virginia.

*Harvest Season:* Late July- Mid October

*Estimated Annual Inventory:* 27,958,773 ton


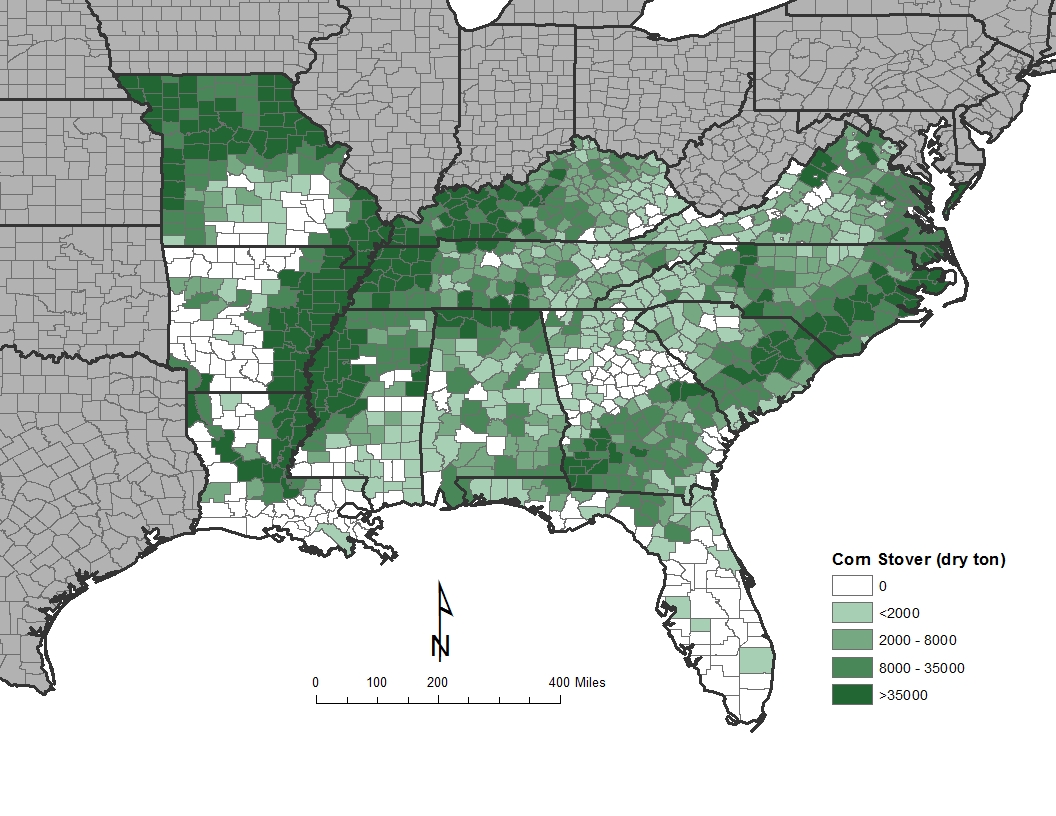


Figure 1. Annual Corn Stover Inventory in the Southeastern U.S. (USDA, 2014)

*Southwestern U.S:* Corn production in the southwestern U.S. is distributed across the region with areas of high production located in the coastal plain of Texas, the panhandles of Texas and Oklahoma, eastern Colorado, western Utah and the central valley in California.

*Harvest Season:* Mid August – Early November

*Estimated Annual Inventory:* 11,187,082 ton

*
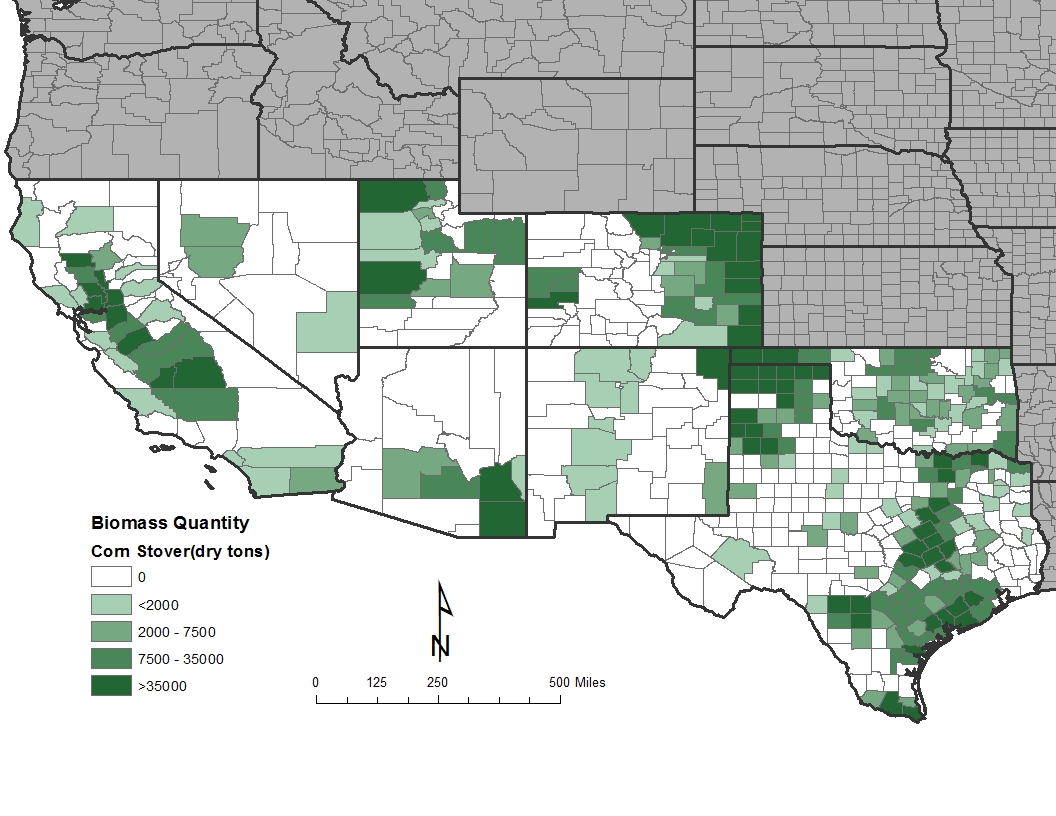
*

Figure 2. Annual Corn Stover Inventory in the Southwestern U.S. (USDA, 2014)

***Cotton Stalks***

Cotton stalks are an unused crop residue that may have potential as a bioenergy feedstock. Historically, the stalks have been destroyed and tilled back into the soil. The purpose for this is two-fold, first is to control pest and disease and second to improve the soil in the field(Hake, Carter et al. 1991). The primary reason for the destruction of the stalks is to control boll weevils and pink bollworms, both potentially destructive to the future cotton crop. Additionally, returning the stalks to the soil replaces nutrients and provides some benefit in soil preparation. Removing the stalks from the field , could play the same role as destroying the stalks in pest management and the minor loss of nutrients could be made up with application of fertilizer.

*Southeastern U.S.:* Cotton production in the southeastern U.S. is centered around two primary locations. The first location is the Mississippi River valley from southern Missouri to central Louisiana. The second location is the coastal plain from southern Alabama and the Florida panhandle to Virginia.

*Harvest Season:* Late September – Mid November

*Estimated Annual Inventory:* 4,174,541 ton


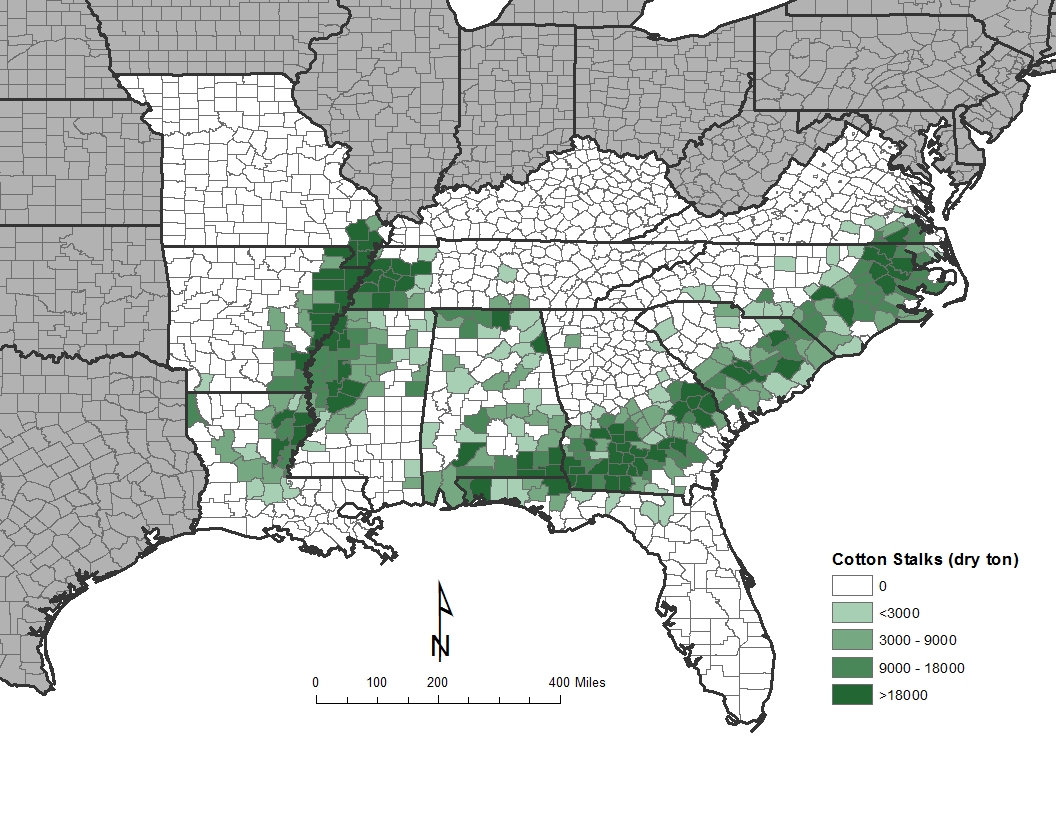


Figure 3. Annual Cotton Stalk Inventory in the Southeastern U.S. (USDA, 2014)

*Southwestern U.S:* Cotton production in the southwestern U.S. is located primarily in the southern Central Valley of California and Southern Arizona, with an area of high production in the Texas panhandle and Gulf coast areas.

*Harvest Season: Mid October – Early December*

*Estimated Annual Inventory:* 4,064,226 ton

***
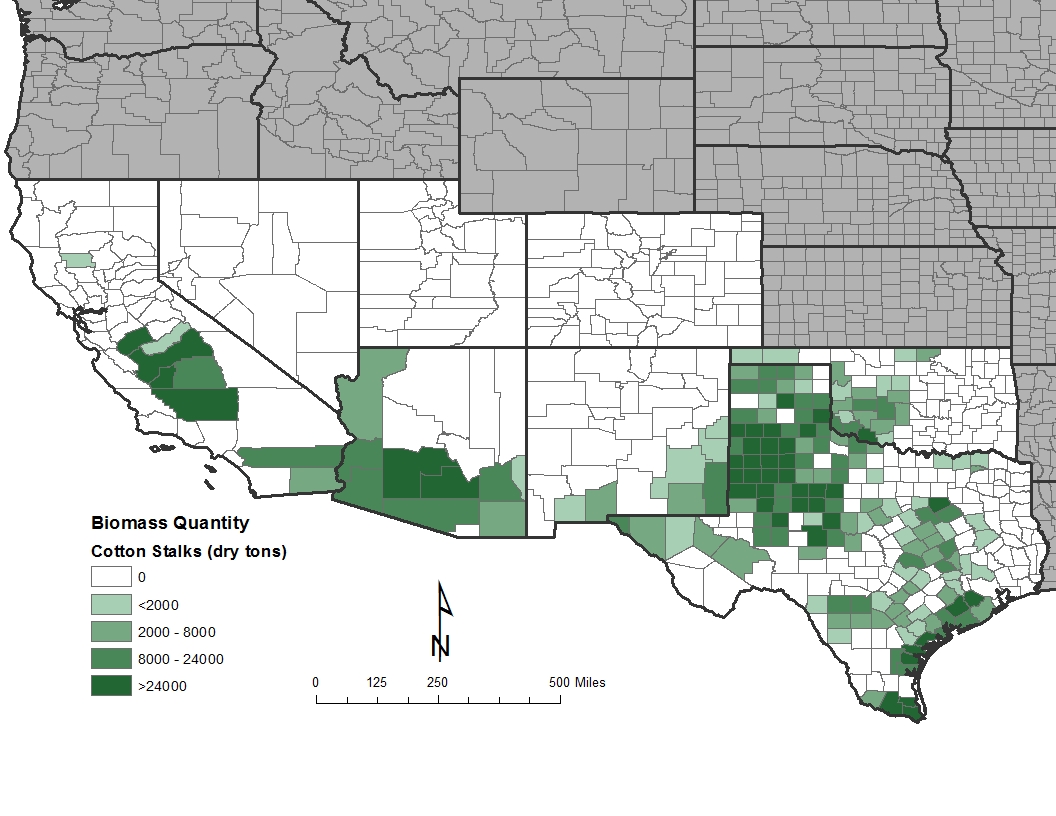
***

Figure 4. Annual Cotton Stalk Inventory in the Southwestern U.S. (USDA, 2014)

***Peanut Hay***

Peanut hay is the residue of the peanut production. Peanuts are an import oil and food crop that is cultivated in the tropical and warm temperate regions of the world. While the production of peanuts is primarily for food and oil production, the seeds are also utilized in the production of soaps, medicine and cosmetics. The residues from peanut production are primarily utilized as fodder for horses and other livestock (Putnam et al., 1991).

*Southeastern U.S.:* Peanut hay inventory in the southeastern U.S. (Figure 5) is primarily limited to the coastal plain stretching from Alabama to Virginia, with areas of the highest production in southern Georgia.

*Harvest Season:* Mid September – Late October

*Estimated Annual Inventory:* 4,383,210 ton


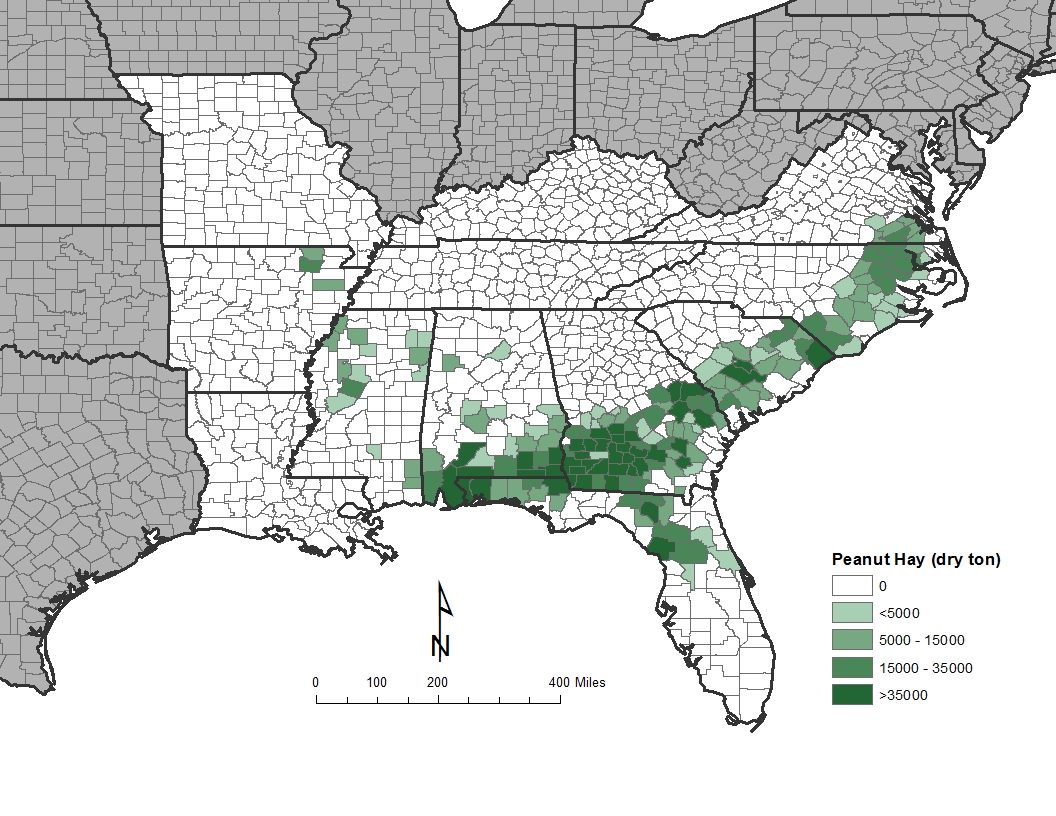


Figure 5. Annual Peanut Hay Inventory int the Southeastern U.S. (USDA, 2014)

*Southwestern U.S:* Peanut production in the southwestern U.S. (Figure 6) is distributed throughout Oklahoma, New Mexico and Texas. The areas with the highest concentration of inventory are the southwestern Oklahoma – Texas border, the southeastern New Mexico – Texas border and southcentral Texas.

*Harvest Season:* Mid October – Mid November

*Estimated Annual Inventory:* 462,923 ton

*
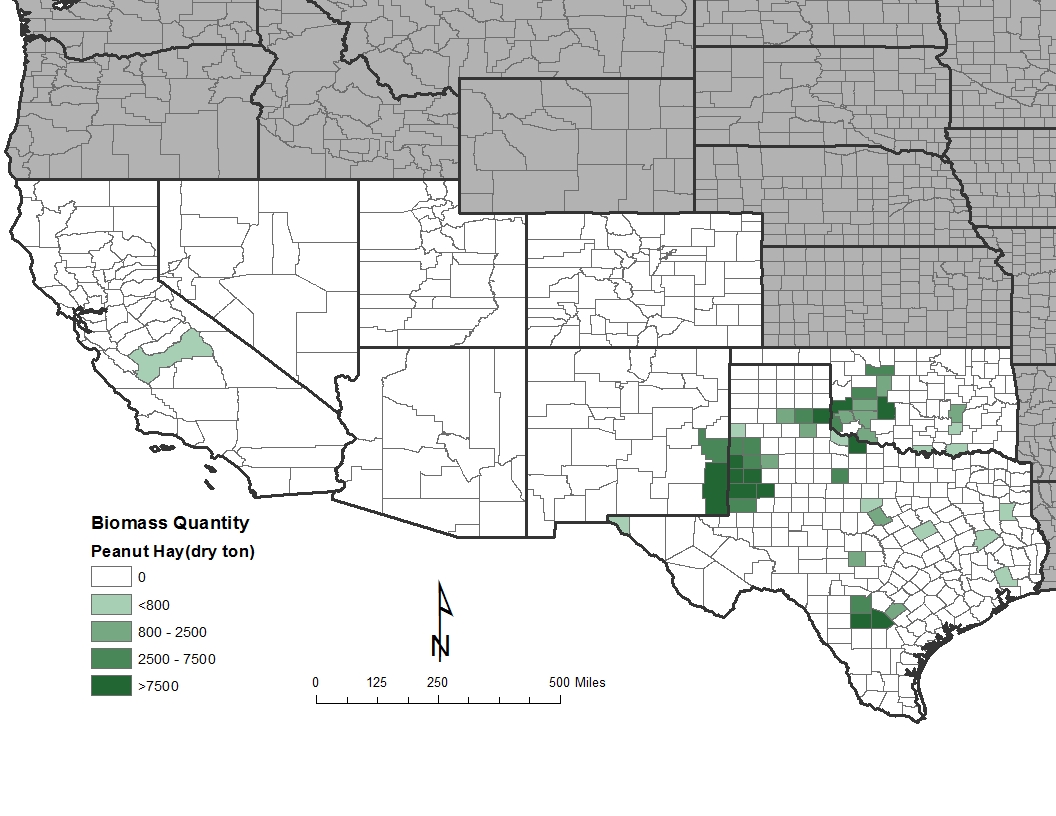
*

Figure 6. Annual Peanut Hay Inventory in the Southwestern U.S. (USDA, 2014)

***Rice Straw***

Rice straw is the residue that remains from rice production. While rice production in the U.S. is minor compared to the production of Asia, the quantity of rice straw produced may be potential resource for energy production (Kadam, Forrest and Jacobson 2000). Rice straw constitutes between 40 – 60% of the dry weight of the rice plant and for each ton of grain produced approximately 1.35 tons of straw is produced. Currently, due to there being few uses and residual straw interferes with planting implements the straw is burned in the field.

*Southeastern U.S.:* Rice straw inventory in the southeastern U.S.(Figure 7) is almost entirely located in the Mississippi river valley from Missouri to Louisiana and in the Mississippi river delta in south west Louisiana.

*Harvest Season:* Late August – Mid October

*Estimated Annual Inventory:* 7,157,144 ton


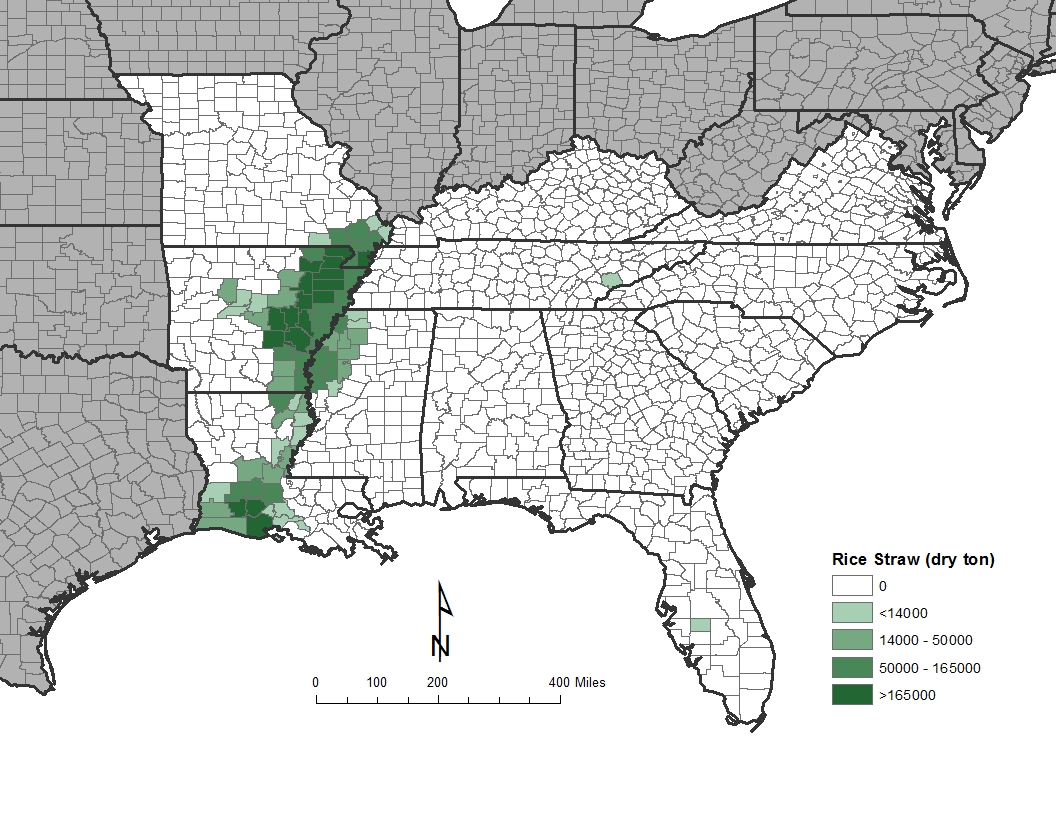


Figure 7. Annual Rice Straw Inventory in the Southeastern U.S. (USDA, 2014)

*Southwestern U.S:* Rice straw inventory in the southwestern U.S.(Figure 8) is found in the northern central valley of California and along the Gulf Coast of Texas.

*Harvest Season:* Early October (California)

Mid August – Mid September (Texas)

*Estimated Annual Inventory:* 2,756,610 ton

*
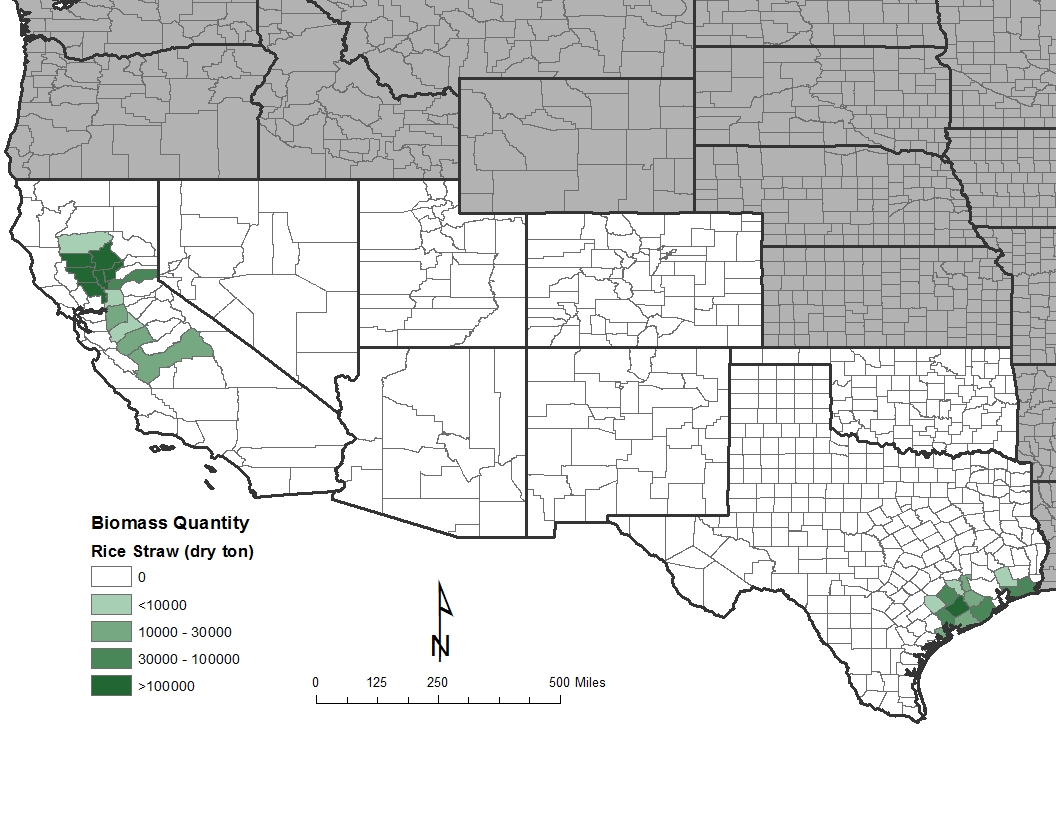
*

Figure 8. Annual Rice Straw Inventory in the Southwestern U.S. (USDA, 2014)

***Sorghum***

Sorghum is a tropical annual grass that is quick growing and is able to be grown in a wide ranging areas ranging from tropical to temperate and semi-arid locations. Additionally, sorghum generally requires less fertilizer than corn and can tolerate a variety of soil conditions. There are primarily four types of sorghum: grain, forage, high-tonnage and sweet(Shoemaker and Bransby 2010). Grain, forage and high-tonnage sorghums are primarily used to feed livestock domestically, as either grazing, hay or silage material. Sweet sorghum on the other hand has been primarily used for human consumption, in which the high sugar content juice from the plant is use to make a molasses substitute. The range for sorghum production is primarily in the Great Plains, but can be grown profitably in practically all locations of the U.S.

*Southeastern U.S.:* Sorghum inventory in the southeastern U.S. (Figure 9) is distributed across the region, with production of varying levels occurring in each state.

*Harvest Season:* Mid August – Late October

*Estimated Annual Inventory:* 776,201 ton

*
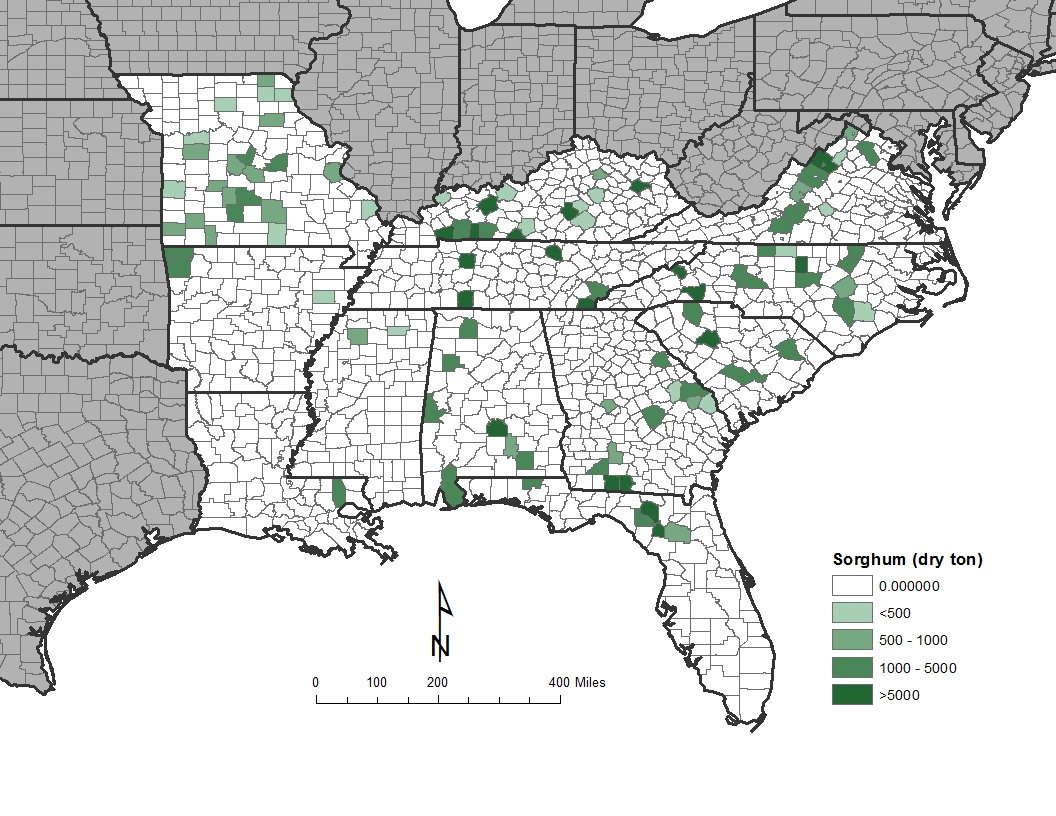
*

Figure 9. Annual Sorghum Inventory in the Southeastern U.S. (USDA, 2014)

*Southwestern U.S:* Sorghum inventory in the southwestern U.S. (Figure 10) is spread across the region with areas of localized high production in the lower central valley of California, southern Arizona, and the eastern New Mexico border– Texas Panhandle.

*Harvest Season:* Early August – Early October

*Estimated Annual Inventory:* 1,256,652 ton

***
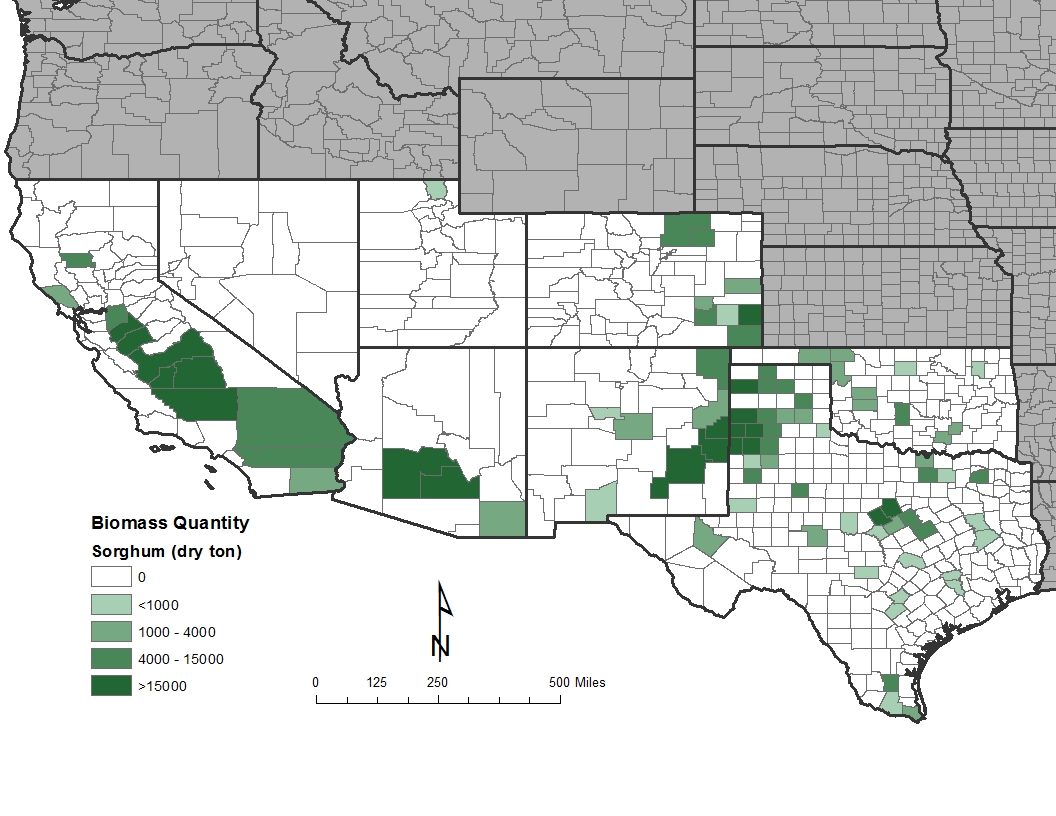
***

Figure 10. Annual Sorghum Inventory in the Southwestern U.S. (USDA,2014)

***Haylage***

Haylage is a method of processing and storing grass and legume crops that are traditionally stored as hay at a moisture content of 40 – 60%. Due to the higher moisture content the materials must be stored anaerobic conditions, or there is risk of excessive heating and mold growth. The current market for haylage is as fodder for livestock. Haylage is preferable to hay for feeding livestock because a higher proportion of the nutrients are retained and there are lower levels of field losses. It is for these same reasons that haylage is being considered as a bioenergy feedstock.

*Southeastern U.S.:* Haylage inventories in the southeastern U.S. (Figure 11) are fairly uniformly distributed across the region with conspicuous lower inventory levels in Arkansas and southern Georgia.

*Harvest Season:* Late April – Early September

*Estimated Annual Inventory:* 934,803 ton

*
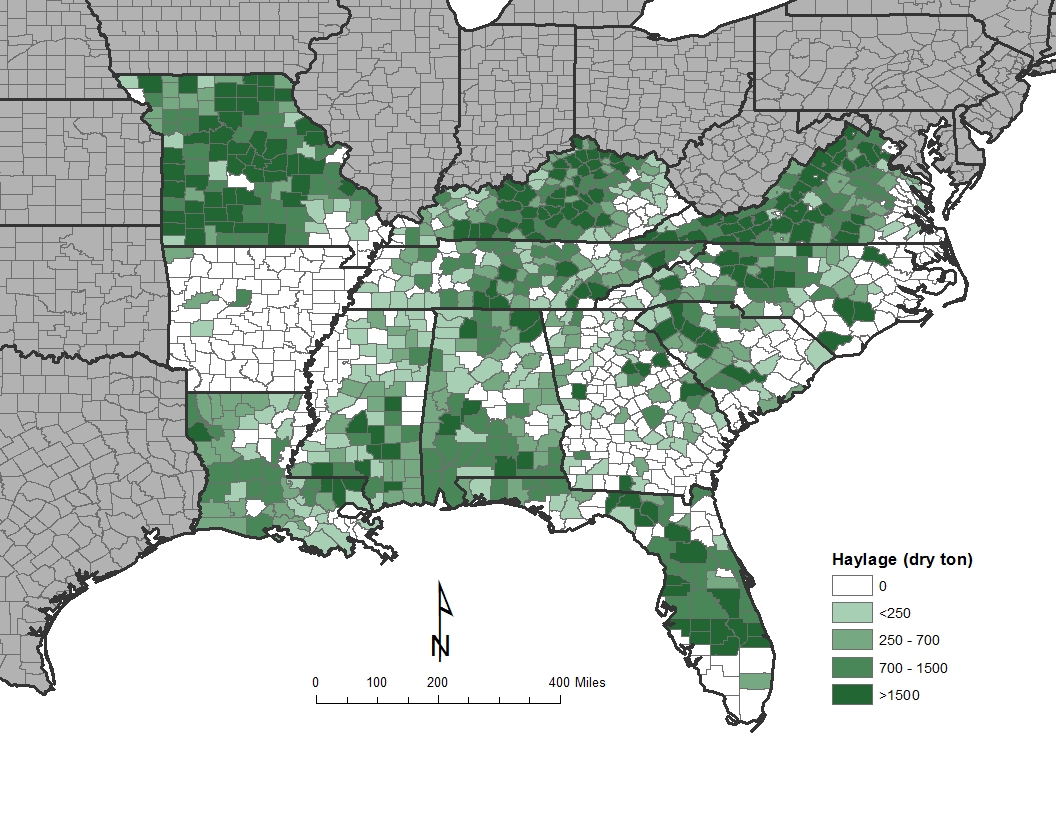
*

Figure 11. Annual Haylage Inventory in the Southeastern U.S. (USDA, 2014)

*Southwestern U.S:* Haylage inventories in the southwestern U.S. (Figure 12) show that there is material available widely across the region while there are localized areas of no production.

*Harvest Season:* Mid April- Early September

*Estimated Annual Inventory:* 2,866,954 ton

***
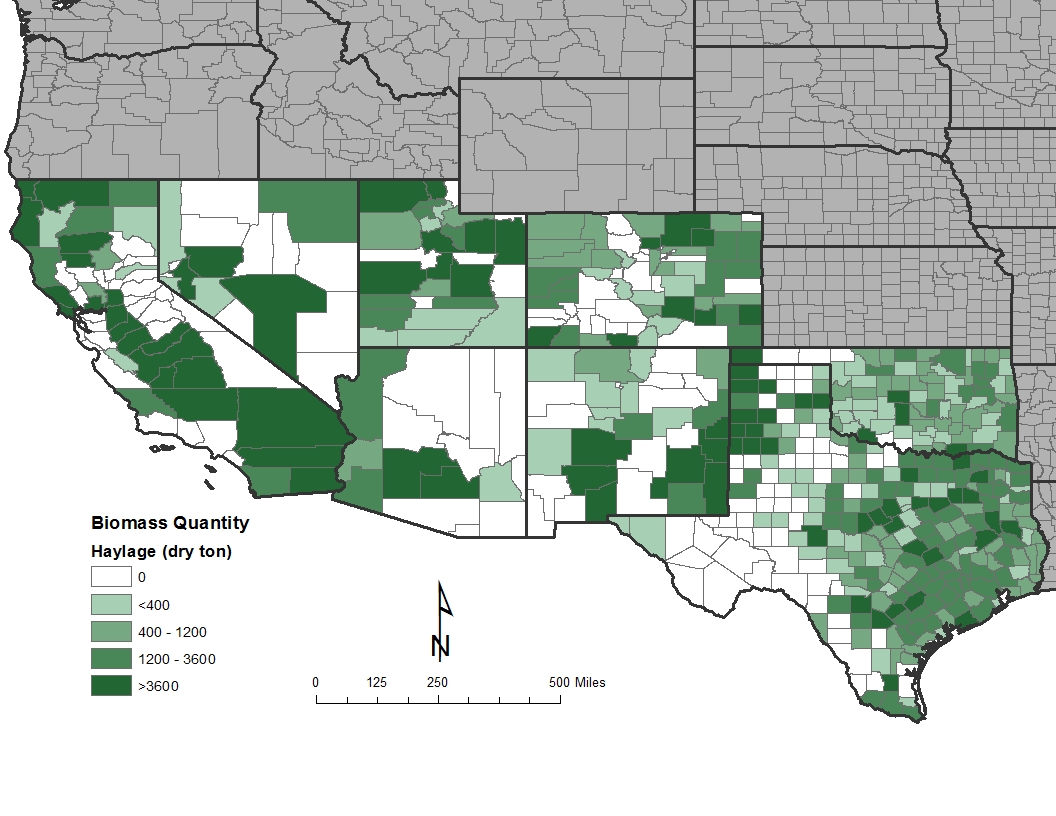
***

Figure 12. Annual Haylage inventory in the Southwestern U.S. (USDA, 2014)

***Distillers Grains***

Dried distillers grains are a byproduct of the distillation process and are produced by either traditional brewers and distillers making alcohol for consumption or more recently by the production ethanol fuel. Distillers grains are typically produced as Wet Distillers Grains or Dried Distillers Grains. Wet Distillers Grains are up to 70% moisture and are highly susceptible to mold and degradation after 4 – 5 days (Weigel, Loy et al. 1997). Dried Distillers Grains are only 10 – 12% moisture and have an almost indefinite shelf life (Weigel, Loy et al. 1997). Currently the most common use for distillers grains is as a high protein animal feed.

*Southeastern U.S.:* Inventories of Distillers Grains in the southeastern U.S. (Figure 13) are limited to the locations of facilities that process corn for fermentation.

*Harvest Season:* Continuous

*Estimated Annual Inventory:* 2,064,000 ton

*
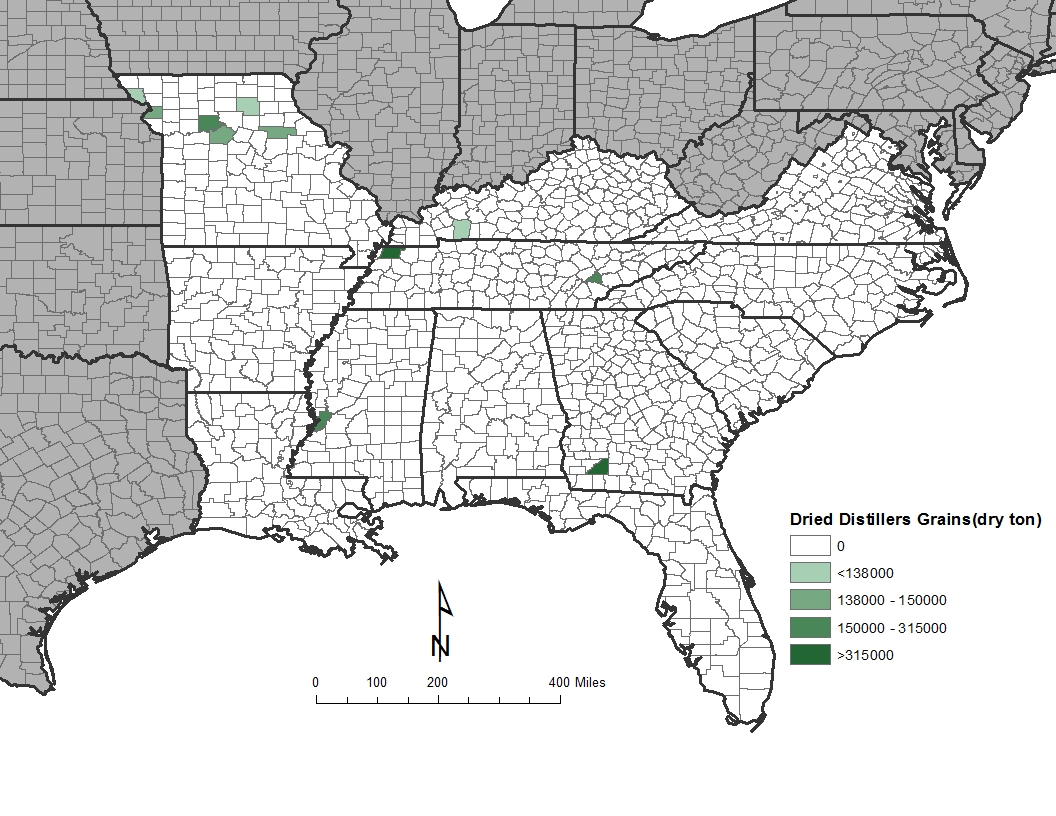
*

Figure 13. Annual Inventory of Distillers Grains in the Southeastern U.S. (Ethanol Producers Magazine, 2016)

*Southwestern U.S:* Inventories of Distillers Grains in the southwestern U.S. (Figure 14) are limited to the locations of facilities that process corn for fermentation.

*Harvest Season:* Continuous

*Estimated Annual Inventory:*2,496,000 ton

***
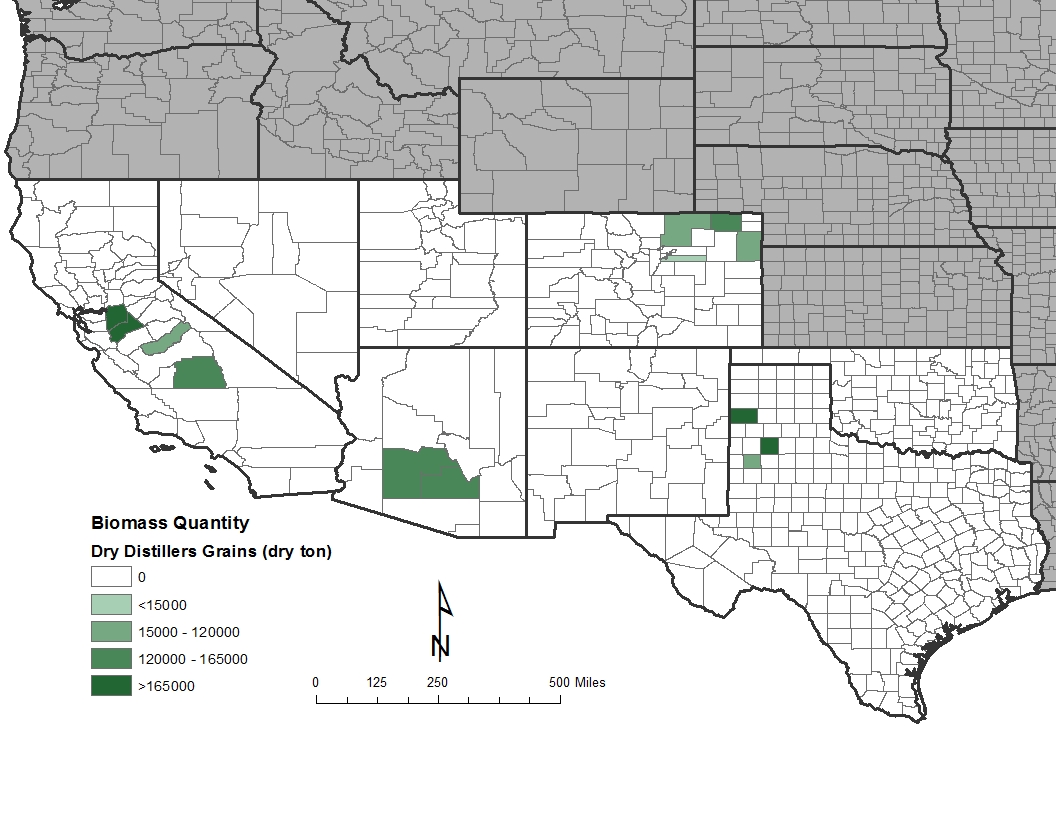
***

Figure 14. Annual Inventory of Distillers Grains in the Southwestern U.S. (Ethanol Producers Magazine, 2016)

***Sugar cane/Energy cane***

Sugar cane is a feedstock that is rich in sucrose and is one of the main sources of sugar production globally (Matsuoka, Kennedy et al. 2014). In addition to the sugar yield, sugar cane also produces a high proportion of fibrous residue in the form of bagasse. It is estimated that approximately 30% of the plant weight remains as bagasse after sugar extraction (Austin 2011). Due to the apparent potential of the production of fiber from sugar cane, plant breeding has been used to modify sugar cane to produce higher per acre yields and a greater amount of fiber. These new cultivars are termed energy cane. While the energy cane has higher per acre yield and higher amounts of fiber, the sucrose levels have been decreased(Carvalho-Netto, Bressiani et al. 2014).

*Southeastern U.S.:* Current inventory of Sugar Cane/Energy Cane in the southeastern U.S. (Figure 15) is limited to the Mississippi River Delta in southern Louisiana and southern Florida.

*Harvest Season:* Late October – Early March

*Estimated Annual Inventory:* 27,380,199 ton

*
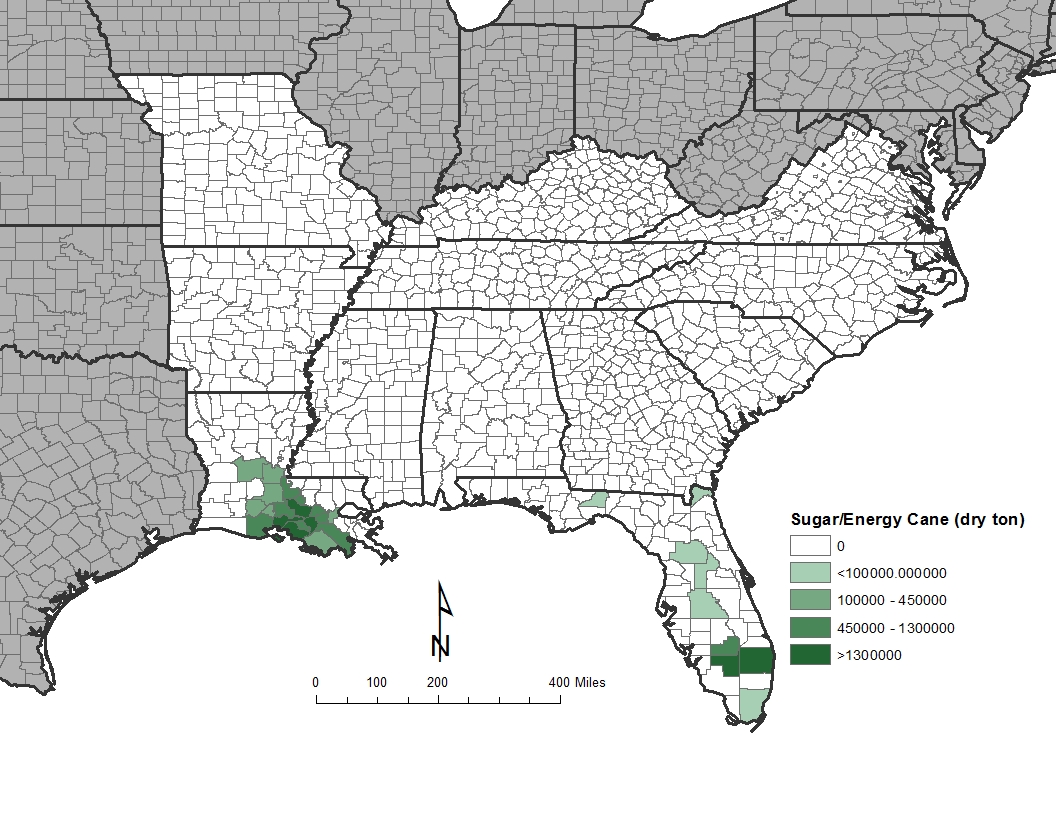
*

Figure 15. Annual Inventory of Sugar Cane/Energy Cane in the Southeastern U.S. (USDA, 2014)

*Southwestern U.S:* Current inventory of Sugar Cane/Energy Cane in the southwestern U.S. (Figure 16) is limited to a extremely small region in the southern Gulf Coast of Texas.

*Harvest Season:* Early November – Early March

*Estimated Annual Inventory:*1,401,926 ton

***
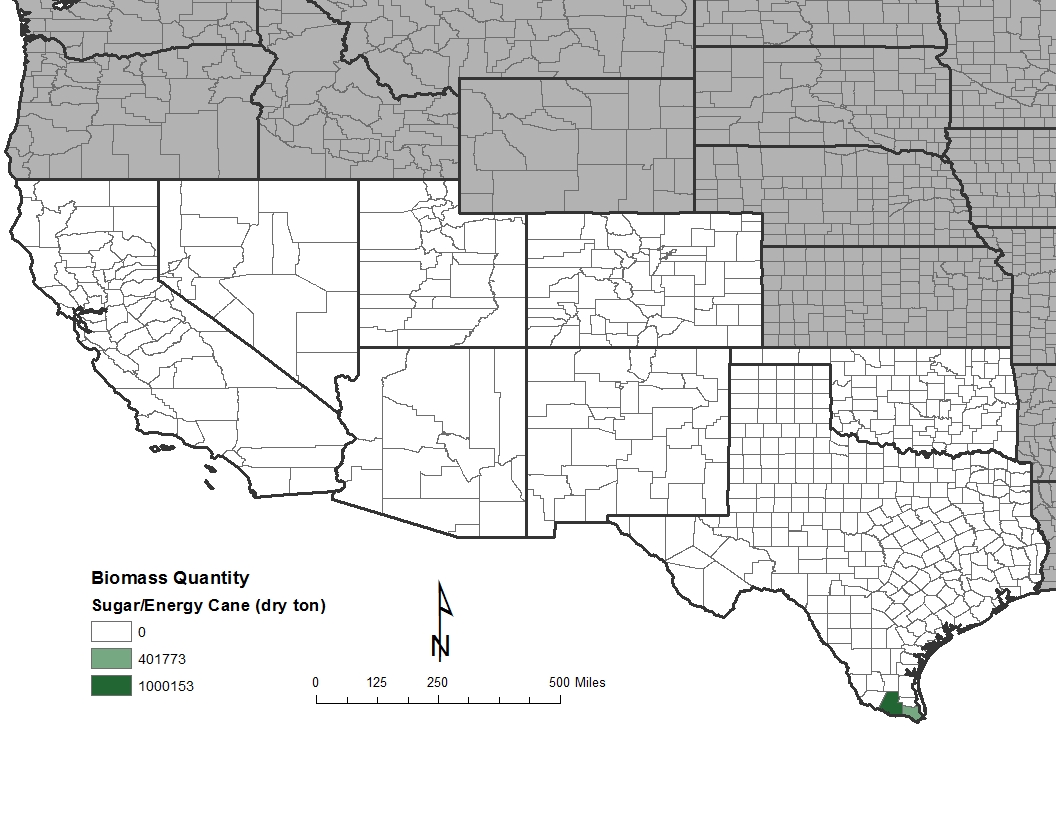
***

Figure 16. Annual Inventory of Sugar Cane/Energy Cane in the Southwestern U.S. (USDA, 2014)

***Yard waste***

Yard waste is the green vegetative organic matter that results from the maintenance of yards, parks and other public and private green space. Yard waste has historically been disposed of into landfills, which causes a problem as the material biodegrades. As the vegetation biodegrades, biogas which contains methane, a highly potent greenhouse gas, is produced and released into the atmosphere. Yard wastes are produced in practically all areas of the contiguous United States, and are a potentially low cost bioenergy feedstock.

*Southeastern U.S.:* The estimated inventory of yard waste in the southeastern U.S. (Figure 17) is distributed across the region with clear areas of higher inventories that coincide with areas of higher population

*Harvest Season:* Late April – Late September

*Estimated Annual Inventory:* 2,439,955 ton

*
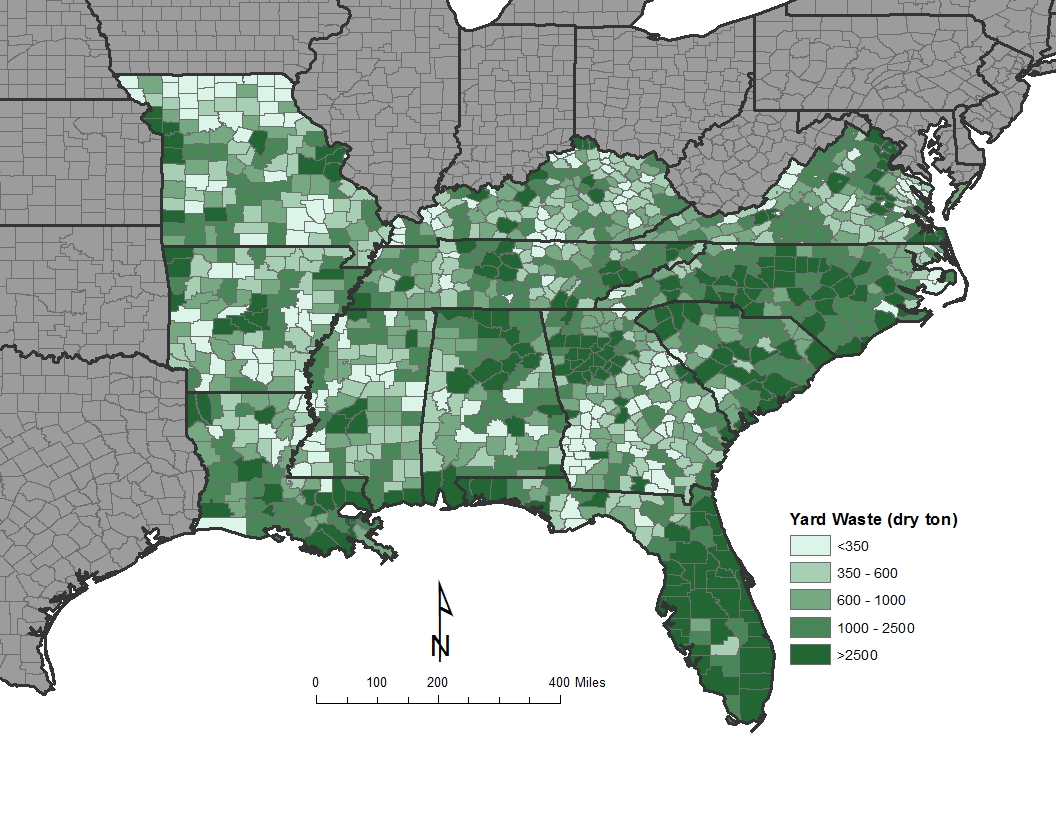
*

Figure 17. Annual Inventory of Yard Waste in the Southeastern U.S.

*Southwestern U.S:* Similar to the inventory of the of yard waste in the southeastern U.S.; the inventory of yard wastes in the southwestern U.S. are distributed across the region and shows areas of higher inventory where the population increases. However, it is also apparent that the southwest U.S. also has larger contiguous areas of lower population that result in lower estimates of yard waste.

*Harvest Season:* Late April – Late September

*Estimated Annual Inventory:* 2,588,903 ton

*
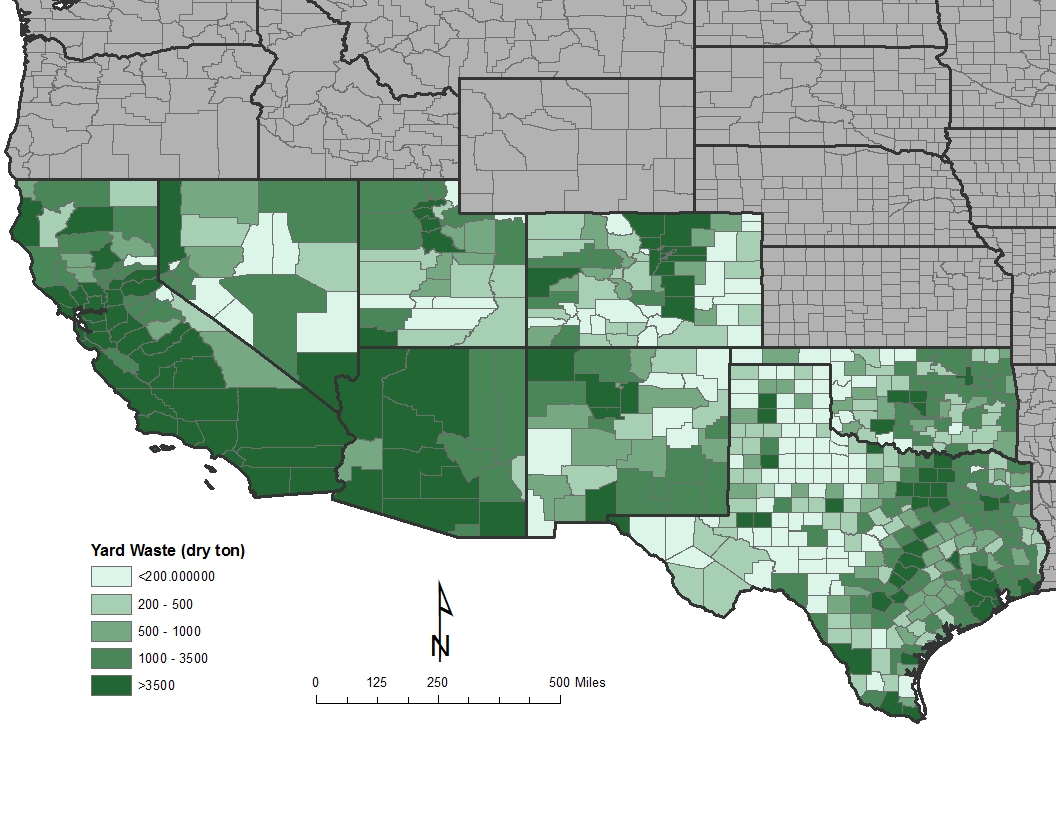
*

Figure 18. Annual Inventory of Yard Waste in the Southwestern U.S.

(2011). West Virginia Solid Waste Management Plan, 2011, West Virginia Solid Waste Management Board.

Abramowitz, H. and Y. Sun (2012). Municipal Solid Waste Characterization Study for Indiana, Purdue University Calumet.

Austin, A. (2011). Bagasse Power. Biomass Grand Forks, ND, BBI International.

Carvalho-Netto, O. V., J. A. Bressiani, H. L. Soriano, C. S. Fiori, J. M. Santos, G. V. Barbosa, M. A. Xavier, M. G. Landell and G. A. Pereira (2014). "The potential of the energy cane as the main biomass crop for the cellulosic industry." Chemical and Biological Technologies in Agriculture **1**(1): 1-8.

Cascadia Consulting Group (2008). California 2008 Statewide waste characterization study.

Cascadia Consulting Group (2010). 2009 Washington Statewide Waste Characterization Study, Dept of Ecology, State of Washington.

Cascadia Consulting Group, DSM Environmental Services and MSWConsultants (2007). Delaware solid waste authority statewide waste characterization study, 2006-2007.

Cascadia Consulting Group, R.W. Beck and GRG Analysis (2003). Wisconsin Statewide Waste Characterization Study, Wisconsin Department of Natural Resources.

Cascadia Consulting Group, Sky Valley Associates and R. W. Beck (2003). Characterization of wastes from single-family residences.

CDM (2009). Illinois Commodity/Waste Generation and Characterization Study, Illinois Department of Commerce and Economic Opportunity.

Covanta Energy SEMASS and Mid Atlantic Solid Waste Consultants (2010). "Waste Characterization Study in Support of Class II Recycling Program."

Covanta Energy Springfield and Mid Atlantic Solid Waste Consultants (2010). "Waste Characterization Study in Support of Class II Recycling Program."

Criner, G. K. (2012). "2011 Maine Residential Waste Characterization Study."

DSM Environmental Services (2002). Vermont Waste Composition Study, Vermont Department of Environmental Conservation, Solid Waste Program.

Engineering Solutions and Design, I. (2004). State of Ohio Waste Characteriaztion Study, Ohio Department of Natural Resources, Division of Recycling and Litter Prevention.

Engineering Solutions and Design, I. (2009). State of Nebraska Waste Characterization Study, Nebraska Department of Environmental Quality.

Ethanol Producer Magazine (2016). "Fuel ethanol industry directory." <https://issuu.com/bbiinternational/docs/feid-2016>.

Florida Department of Environmental Protection (2010). Florida Municipal Solid Waste Collected and Recycled 2010, Florida Department of Environmental Protection.

Franklin Associates (2003). Strategic Directions and Policy Recommendations for Solid Waste Management in the Bi-State Kansas City Metropolitan Region, Mid America Regional Council.

Hake, K., L. Carter, L. Moore, R. Parker, R. Summy, T. Watson and R. Williford (1991) "Cotton Stalk Management." Cotton Physiology Today **2**.

Jeschke, M. and A. Heggenstaller (2012) "Sustainable Corn Stover Harvest for Biofuel Production." Crop Insights **22**, 6.

Koopmans, A. and J. Koppejan (1997). "Agricultural and forest residues-generation, utilization and availability." Paper presented at the regional consultation on modern applications of biomass energy **6**: 10.

Matsuoka, S., A. J. Kennedy, E. G. D. d. Santos, A. Tomazela, #xe9, L. and L. C. S. Rubio (2014). "Energy Cane: Its Concept, Development, Characteristics, and Prospects." Advances in Botany **2014**: 13.

Mid Atlantic Solid Waste Consultants (2007). Larimer County, Colorado Two-Season waste composition study.

Mid Atlantic Solid Waste Consultants (2011). Iowa Statewide Waste Characterization Study.

Midwest Assistance Program (2008). 2008 Missouri Waste Composition Study, Missouri Department of Natural Resources, Solid Waste Management Program.

MSWConsultants and Cascadia Consulting Group (2010). 2010 Waste Composition Study, Prepared for Boulder County resource conservation divsion.

NYSDEC. (2010). "Solid Waste Composition and Characterization." from <http://www.dec.ny.gov/chemical/65541.html>.

ODEQ. (2010). "Table A2. Statewide Waste Composition Results." Retrieved 4/11/16, from <http://www.deq.state.or.us/lq/sw/disposal/wastecompstudy2009.htm>.

R.W. Beck (200). Statewid Waste Composition Study, Pennsylvania Departlment of Environmental Protection.

R.W. Beck (2005). Georgia Statewide waste characterization study, Georgia Department of Community affairs.

RIDOP (2005). Rhode Island Comprehensive Solid Waste Management Plan, Rhode Island Divison of Planning.

SCS Engineers (2010). Montgomery County, Maryland Waste Composition Sampling & Analysis Study, Montgomery County Department of Environmental Protection**:** 51.

SDM Envrionmental Services, Cascadia Consulting Group and Mid Atlantic Solid Waste Consultants (2010). Connecticut State Wide Solid Waste Composition and Characterization Study.

Shoemaker, C. E. and D. I. Bransby (2010). The role of sorghum as a bioenergy feedstock. Sustainable alternative fuel feedstock opportunities, challenges and roadmaps for six US regions, Proceeedings of the Sustainable Feedstocks for Advance Biofuels Workshop, Atlanta, GA.

TCEQ (2006). Municipal Solid Waste in Texas: A Year in Review 2006 Data Summary and Analysis, Texas Commission on Environmental Quality, Waste Permits Division.

Vilsack, T. (2014). United States Department of Agriculture, 2012 Census of agriculture.

Weigel, J. C., D. Loy, L. H. Kilmer, I. C. G. Association, R. F. Association and N. C. G. Association (1997). Feed co-products of the corn wet milling process, Iowa Corn Growers Association.

Weigel, J. C., D. Loy, L. H. Kilmer, I. C. G. Association, R. F. Association and N. C. G. Association (1997). Feed co-products of the dry corn milling process, Iowa Corn Growers Association.
